# Medico-Legal Intelligence

**Published:** 1897-06-26

**Authors:** 


### MEDICO-LEGAL INTELLIGENCE.

The following report, which has been sent to us by the solicitors to the London and Counties Medical Protection Society, will be read with interest by many practitioners: Dr. Joseph John Clarke, of Markhouse Road, Walthamstow, who ordered an injured woman into hospital, and when she died gave evidence at the inquest in response to a summons, sued the coroner, Mr. Charles Edgar Lewis, at Brentwood County Court, to obtain a guinea for his remuneration. Plaintiff was represented by a barrister, Mr. W. Ellis Hill, instructed by Messrs. Le Brasseur and Oakley, the solicitors to the London and Counties Medical Protection Society (Limited), and the South Essex Coroner, who was defendant, was represented by the South-West London Coroner (Mr. A. Braxton Hicks), who is also a barrister. Mr. Hill said that in the Coroners Act there is a provision that where an inquest was held on the body of a person who had died in a hospital or infirmary used for the reception of patients, the medical officer whose duty it might have been to attend to such deceased person as medical officer of such institution should not be entitled to recover any fee or remuneration. The plaintiff, counsel added, was not the medical officer of the institution. Mr. Hicks resisted the claim because, he said, coroners if they paid fees to these medical men were surcharged by the auditors. His Honour held the plaintiff was not the doctor whose duty it was to attend the deceased person as medical officer of the hospital, and was therefore entitled to his fee as a witness. Judgment was given for plaintiff, with costs on the higher scale. Mr. Hicks said the case would be taken to the High Court, and leave to appeal was given.

